# Identification of Pathogenic Factors in *Klebsiella pneumoniae* Using Impedimetric Sensor Equipped with Biomimetic Surfaces

**DOI:** 10.3390/s17061406

**Published:** 2017-06-15

**Authors:** Duyen Thi Ngoc Huynh, Ah-Young Kim, Young-Rok Kim

**Affiliations:** Department of Food Science and Biotechnology & Graduate School of Biotechnology, College of Life Sciences, Kyung Hee University, Yongin 17104, Korea; htnduyen@gmail.com (D.T.N.H.); jla42@naver.com (A.-Y.K.)

**Keywords:** impedimetric sensor, virulence factors, *Klebsiella pneumoniae*, lipopolysaccharide, fimbriae

## Abstract

*K. pneumoniae* is an opportunistic pathogen that causes nosocomial infections, such as, pneumonia, urinary tract infections, septic shock, and gastro intestinal disease. Lipopolysaccharide (LPS), capsular polysaccharide, and fimbriae are recognized major virulence factors of *K. pneumoniae* and play key roles during early stages of infections. In this study, we functionalized the surface of gold electrode with mannose and mucin to monitor the adhesion-associated virulence factors of *K. pneumoniae*. The binding characteristics of *K. pneumoniae* 2242 wild type and of its isogenic mutants lacking outer-core LPS (∆*wabG*) or fimbriae (∆*fimA*) were investigated using Faradaic impedance spectra. The results obtained showed fimbriae are responsible for *K. pneumoniae* adhesion to the mannose of glycoprotein on the surfaces of epithelial cells, whereas outer-core LPS and capsular polysaccharide are associated with specific binding to mucous. These results concurred with those of a conventional in vitro assay using human ileocecal epithelial cell (HCT-8 cells) and a human bladder epithelial cell (T-24), indicating that the devised method could be useful for investigating virulence-associated interactions of pathogenic bacteria with specific host cells and organs.

## 1. Introduction

*K. pneumoniae* is a well-known opportunistic pathogen and responsible for ~10% of nosocomial bacterial infections, which include sepsis, pneumonia, urinary tract infections, and hepatic abscess [1,2,3]. The bacterium can infect almost every part of the human body, although the urinary and respiratory tracts are most commonly affected [4]. The pathogenic factors of *K. pneumoniae* have been determined to be capsular polysaccharide (CPS), lipopolysaccharide (LPS), and fimbriae [5]. LPS is a major component of the outer membrane of Gram-negative bacteria, and consists of lipid A, core oligosaccharide, and a long chain polysaccharide (O-antigen). LPS is an important pathogenic determinant in *K. pneumoniae*, which causes pneumonia and bacteremia in man. O-antigen is responsible for bacterial resistance to complement-mediated killing. On the other hand, CPS is considered the most important virulence factor of *K. pneumoniae*. It covers the bacterial surface and is responsible for its resistance to host phagocytes and serum and promotes inflammation and sepsis [5,6,7]. The colonization of mucous membranes by bacteria is the result of adhesion between the bacterial capsule and the host’s mucous layer [5,8]. The adhesion of *K. pneumoniae* to mammalian tissue is mediated by two types of bacterial pili, type 1 and type 3 [9,10]. Type 1 fimbriae play an important role in bacterial adhesion to the d-mannose moiety on mammalian cell surfaces due to the specific affinity of fimbrial protein at the tips of fimbriae [11,12]. Type 3 fimbriae are characterized by their affinities for a range of mammalian cells, which include bladder epithelial cells, uroepithelial cells, and endothelial cells [13,14]. The factors present on the surfaces of epithelial cells are recognized by pathogenic bacteria and include certain proteins, glycolipids, and carbohydrates [15]. In particular, carbohydrate-protein recognition plays a key role in most pathogenic processes, including bacterial infections, cancer cell differentiation, and inflammation, and is involved with specific carbohydrate sequences on host cell surfaces [16]. Herein, we describe an impedimetric sensor equipped with a functionalized electrode, which mimics the surfaces of epithelial cells and the luminal surfaces of epithelial organs, and its use to identify the virulence factors of *K. pneumoniae* associated with the adhesion process during the early stage of infection. Outer-core LPS and fimbriae of *K. pneumoniae* are major virulence factors and are known to be responsible for its adhesion to the mucous layer and epithelial cells, respectively [17,18]. The mucins, which are major components of mucous layers, compose a family of glycoproteins that cover the luminal surfaces of epithelial organs [19]. On the other hand, the mannose moiety of N-linked glycoprotein on the outer surfaces of human epithelial cell membranes has been reported to be associated with the specific interaction with fimbriae of *K. pneumoniae* [20]. We functionalized the surface of a gold electrode with mucin and mannose, and examined its ability to monitor the presence of outer-core LPS and fimbriae of *K. pneumoniae*. In this study, we employed *K. pneumoniae* 2242 wild type and isogenic mutants devoid of outer-core LPS and fimbriae. The changes in Faradaic impedance spectra derived from direct interaction between the bacterium and the model surface were analyzed to monitor the presence of virulence factors. Conventional in vitro assays using human ileocecal epithelial cells (HCT-8 cells) and human bladder epithelial cells (T-24 cells) were performed to validate the accuracy of the developed impedimetric sensing system.

## 2. Materials and Methods 

### 2.1. Materials

Mucin (from porcine stomach, Type III), 11-mercapto-1-undecanol, epichlorohydrin, diethylene glycol dimethyl ether, potassium chloride, potassium hexacyanoferrate (II) trihydrate, potassium ferricyanide (III), sodium phosphate, and d-mannose were purchased from Sigma-Aldrich (St. Louis, MO, USA). Absolute ethyl alcohol, sodium hydroxide, acetone, sulfuric acid, sodium acetate, acetic acid, and hydrogen peroxide were from DaeJung Chemicals & Metals Co., Ltd. (Gyeonggi-do, Korea). Carboxyfluorescein diacetate (CFDA) was obtained from Dojindo Molecular Technologies, Inc. (Rockville, MD, USA), and potassium phosphate from Pure Chemicals Co., Ltd. (Kyoto, Japan). Lysogeny broth (LB) broth and agar powder were purchased from BD (BD Difco, Lawrence, KS, USA). 

### 2.2. Bacterial Strains, Plasmids and Growth Conditions

*Klebsiella pneumoniae* KCTC 2242 (2242) was obtained from the Korean Collection for Type Cultures (Daejeon, Korea). *K. pneumoniae* KCTC 2242 ∆*wabG* mutant strain and *K. pneumoniae* KCTC 2242 ∆*fimA* mutant strain were constructed by Jung et al. [21] and Huynh et al. [17]. For complementation studies, the *wabG* and *fimA* genes were cloned into pBBR1MCS5 vector (CABRI Consortium, Genova, Italy) as follows. The two genes were amplified from *K. pneumoniae* KCTC 2242 by PCR using the following primer sets: for the *wabG* gene; 5′ AAA AAG CTT ATG AGT AAA TTC AGG CTG GC 3′ and 5′ AAA GGA TCC TCA GTG GTG GTG GTG GTG GTG TTA ATT CAC CAG ATC 3′; and for the *fimA* gene; 5′ AAA AAG CTT ATG AAA ATC AAA ACA CTG GC 3′ and 5′ AAA GGA TCC TCA GTG GTG GTG GTG GTG GTG TTA CTC GTA CTG CAC 3′. Amplified *wabG* and *fimA* genes were purified and digested with *Hind*III and *Bam*H I. pBBR1MCS5 vector was also digested with these restriction enzymes. Digested *wabG* and *fimA* genes were ligated into pBBR1MCS5 vector using T4 ligase, and the resulting constructs, pBBR1MCS5::*wabG* and pBBR1MCS5::*fimA* vectors, were transferred into *K. pneumoniae* KCTC 2242 ∆*wabG* mutant and *K. pneumoniae* KCTC 2242 ∆*fimA* mutant, respectively, by electroporation. The complement strains, *K. pneumoniae* KCTC 2242 ∆*wabG* containing pBBR1MCS5::*wabG* and *K. pneumoniae* KCTC 2242 ∆*fimA* containing pBBR1MCS5::*fimA* were selected in LB agar media containing gentamicin, and subsequently confirmed by colony PCR using the primer sets described above. All bacterial strains were incubated at 37 °C in LB broth or LB agar plates containing 10 μg/mL gentamicin. For binding assays, fresh-cultured bacteria were washed three times with PBS buffer (pH 7.4) and then serially diluted in fresh PBS buffer. A conventional plate counting method was used to confirm the number of microorganisms in each sample.

### 2.3. Animal Cell Lines and Culture Conditions

The human ileocecal epithelial cell line HCT-8 and the human bladder epithelial cell line T-24 were provided by the Korean Cell Line Bank (Seoul, Korea). Cells were grown in Roswell Park Memorial Institute (RPMI) 1640 medium (Welgene, Gyeongsan, Korea) supplemented with 10% heat-inactivated fetal bovine serum (FBS), penicillin (100 U/mL), and streptomycin (100 mg/mL). For epithelial cell adherence assays, HCT-8 and T-24 cells were seeded at ~10^4^ cells/well in a 96-well plate (polystyrene; clear flat bottoms; Corning, NY, USA) and grown in 5% CO_2_ for 24 h at 37 °C.

### 2.4. Mucin and Mannose Binding Assays

Functionalizations of 96-well plates with mucin or d-mannose were performed as previously described [22,23]. For the mucin binding assay, mucin was dissolved in an acetate buffer (pH 5.0) to a final concentration of 100 μg/mL, and a 96-well plate was incubated with 100 μL/well of a mucin solution at 37 °C for 24 h. Unbound mucin was removed by washing three times with PBS (pH 7.0). For the mannose binding assay, d-mannose was first dissolved in 0.1 M NaOH to a final concentration of 2% (*w/v*) and 100 μL of the solution was added to the wells of a 96-well plate. After incubating at room temperature for 20 h, the plate was washed three times with PBS (pH 7.0) to remove unbound d-mannose.

Overnight cultures of *K. pneumoniae* were harvested by centrifugation, washed three times with PBS, and resuspended in 0.1 M phosphate buffer (pH 8.5). Carboxylfluorescein diacetate (CFDA) (final concentration 2 μg/mL) was added to the bacterial suspension, which was then incubated at 37 °C for 30 min. Labeled bacteria were washed three times with PBS to remove unbound fluorophore, and resuspended in PBS to a final concentration of 10^5^, 10^6^, 10^7^ or 10^8^ colony forming unit (CFU)/mL. These bacterial suspensions (100 μL) were introduced to wells and incubated at 37 °C for 1 h to assess adhesion. After washing microtiter wells three times with PBS, degrees of bacterial binding to mucin or mannose surfaces was determined by fluorescence spectrophotometry (Tecan, Infinite 200, Grodig, Austria) using excitation and emission wavelengths of 485 and 535 nm, respectively.

### 2.5. Functionalization of a Gold Electrode with Mucin and Mannose

A gold electrode was prepared on glass wafers using an UHV E-Beam Evaporator (ULTECH, Daegu, Korea), as we previously described [24]. Piranha solution (sulfuric acid:hydrogen peroxide, 3:1 (*v/v*)) was used to remove organic residues from 4-inch glass wafers. After cleaning, glass wafers were pre-coated with a 300 Å-thick titanium layer to promote the adhesion of Au to a glass substrate, and then the chip with titanium layer was subsequently coated with a 1000 Å layer of high purity gold using the same electron beam evaporator. The gold-coated glass wafers produced was cut into 10 mm × 20 mm pieces with a wafer dicing machine (DISCO, Tokyo, Japan). The surfaces of gold electrode were then thoroughly washed with acetone and ethanol. The functionalization of gold electrode with mucin or mannose were carried out as follows. For mucin functionalization, a freshly prepared gold electrode was immersed in an 80% ethanolic solution containing 5 mM 11-mercapto-1-undecanol for 3 h at room temperature. The resulting gold electrode, now possessing surface hydroxyl groups, was then washed with ethanol, dried under a gentle stream of nitrogen, and immersed in a solution of 0.6 M epichlorohydrin containing 1:1 mixture of 0.4 M diethylene glycol dimethyl ether and sodium hydroxide for 4 h at 25 °C. After through sequential washing with water, ethanol, and water, the surface of the electrode was treated with a 0.1 M NaOH containing 300 mg/mL of mannose at 4 °C for 20 h. To functionalize the surface of gold electrodes with mucin, chips were immersed in acetate buffer (pH 5.0) containing 5 mg/mL of mucin at 4 °C for 20 h. The electrodes were thoroughly washed with distilled water to remove remaining chemicals and stored in distilled water until required.

### 2.6. Electrical Measurement of Bacterial Adhesion to a Model Surface

A gold electrode functionalized with mucin or mannose was fixed in custom-built Teflon jigs and fastened with four clips to prevent leakage. The microfluidic jig had two openings, which served as the test solution inlet and outlet, and a platinum electrode immersed into the solution in the reaction chamber. A 0.5 mL sample was introduced into the chamber formed on the surface of gold electrode at a flow rate of 50 μL/min using a syringe pump (Model 11 plus, Harvard Apparatus, South Natick, MA, USA) and 1/16” ID Ethylene-tetrafluorethylene (ETFE) tubing (Upchurch Scientific, Oak Harbor, WA, USA). The surface of gold electrode was then washed with 0.5 mL of PBS buffer (pH 7.4) to remove unbound bacteria. PBS buffer (pH 7.4) containing the redox probe [Fe(CN)_6_]^3−/4−^ (at a concentration of 2 mM in the reaction chamber) were introduced before taking measurements. Impedance spectrum data were obtained using an electrochemical analyzer VersaSTAT3 (Princeton Applied Research, Oak Ridge, TN, USA) equipped with V3-Studio software (Princeton Applied Research). The impedance data were collected in the frequency range 0.1 to 1 kHz at the formal potential of the [Fe(CN)_6_]^3−/4−^ redox couple. The magnitude of impedance at each frequency point was analyzed by normalized impedance changes with respect to the control (PBS buffer). Normalized impedance change (NIC) values were calculated using the following formula:

(1) $$\mathrm{NIC}=\frac{Z_{sample}\;-Z_{control}}{Z_{control}}$$

where *Z_control_* is the impedance of the control and *Z_sample_* that of the sample containing bacteria. All experiments were repeated three times and averages and standard deviations were calculated.

### 2.7. Competition Assay

The specific affinity of fimbriae toward mannose was tested by monitoring the binding of *K. pneumoniae* KCTC 2242 to mannose surfaces in the presence of free mannose at concentrations of 0.05, 0.25, or 0.5 mM in PBS (pH 7.4). Bacterial cultures grown overnight were centrifuged at 2000 *g* for 1 min at room temperature, and after washing bacterial pellets three times with PBS, 10^7^ CFU/mL bacterial suspensions were pre-incubated with mannose solutions of respective concentrations for 30 min at room temperature. These solutions were then introduced individually into the fluidic chamber containing the mannose-coated electrode. All experiments were performed three times.

### 2.8. Statistical Analysis

One-way analysis of variance (ANOVA) with Duncan post hoc test was used to determine the significances of differences between the wild-type and mutant types. *p* Values of < 0.05 were considered to denote statistical significance.

## 3. Results and Discussion

### 3.1. Characterization of Model Surface

The adhesion of pathogenic bacteria to human epithelial cells is the first step of infections that could advance to a series of systematic diseases. Therefore, the ability of a bacterium to bind to specific epithelial cells is currently regarded as the key factor for its pathogenicity. However, several reports indicate the ability of *K. pneumoniae* to adhere to cultured epithelial cells is not always associated with bacterial infectivity or virulence, for example, an in vitro study showed no correlation with in vivo results in a mouse model [25]. The reason for this lack of correlation may be that epithelial cells in lung, intestinal tract, and bladder are typically covered with a mucous layer, which serves as physical barrier between the extracellular milieu and the plasma membrane. Furthermore, this mucous layer also facilitates the mucocilliary clearance of potential infectious bacteria from organ surfaces. Accordingly, in vitro studies should be accompanied by in vivo studies to determine the virulence factors or pathogenicity of given bacteria, as these are associated with binding-mediated infection. In the present study, we developed an electrochemical sensing system that can determine the virulence factors associated with binding-mediated infection using a modified model surface that mimics the surfaces of epithelial cells and mucus.

**Figure 1:**
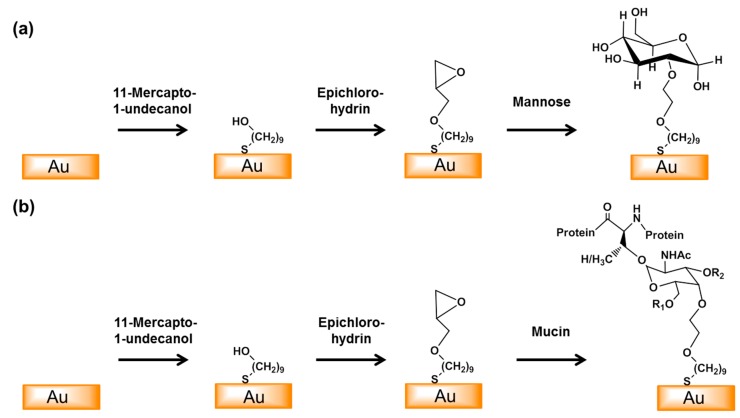
Schematic of the surface modification of the gold electrode with mannose (**a**) or mucin (**b**). An epoxy monolayer was formed by the self-assembly of 11-mercapto-1-undecanol on the surface of the gold electrode and this was then reacted with epichlorohydrin. Mannose and mucin were then coupled to the surface by epoxy ring opening reactions. (R_1_ = β(1,6)-GlcNAc, R_2_ = β(1,3)-GlcNAc).

**Figure 2:**
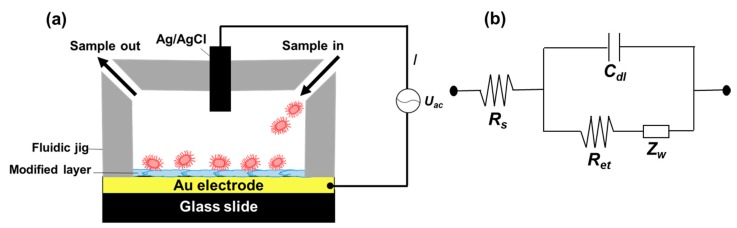
Experiment setup showing the sensing chamber composed of a surface functionalized gold electrode and a Teflon jig (**a**). AC power was connected to a potentiostat that supplied the required current between the functionalized electrode and Ag/AgCl electrode located on top of the fluidic chamber; (**b**) shows a circuit diagram of the set up used for electrochemical impedance spectroscopy measurements in the presence of a redox probe (**b**). The circuit elements shown are; solution resistance (*R_s_*), interfacial electron transfer resistance (*R_et_*), Warbug’s impedance (*Z_w_*), and double layer capacitance (*C_dl_*).

**Figure 3:**
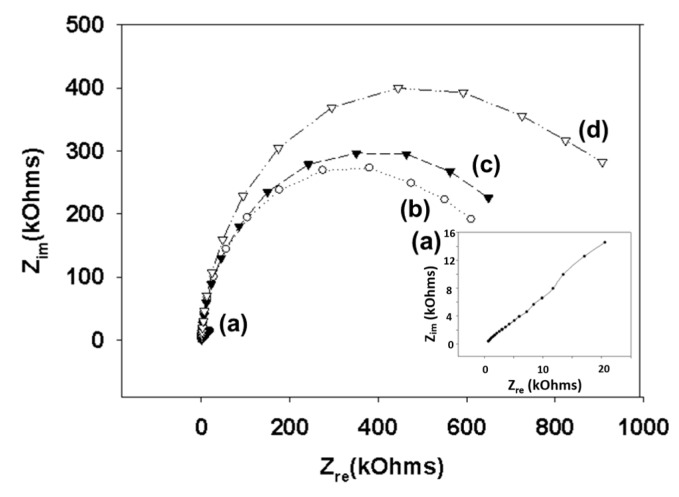
Nyquist diagram (*Z_im_* vs. *Z_re_*) for the Faradaic impedance measurement of a sensing chip in PBS (pH 7.4) containing 2 mM [Fe(CN)_6_]^3−/4−^ over the course of the surface functionalization reaction. A gold electrode (**a**) was treated with 11-mercapto-1-undecanol (**b**), epichlorohydrin (**c**), and mucin (**d**). Impedance spectra were measured within the frequency ranging from 0.1 to 1000 Hz at the formal potential of the [Fe(CN)_6_]^3−/4−^ redox couple. The amplitude of the alternative voltage was 5 mV. The inset shows an enlarged plot for a bare gold electrode.

**Figure 4:**
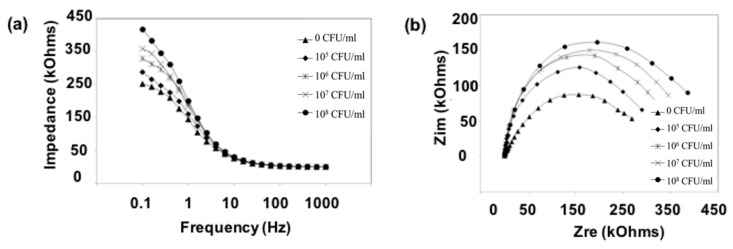
Bode plot (**a**) and Nyquist diagram (**b**) of the Faradaic impedance measurements of a mannose modified electrode in the presence of different concentrations of *K. pneumoniae* KCTC 2242 (from 10^5^ to 10^8^ CFU/mL). Impedance spectra were measured within the frequency ranging from 0.1 to 1 kHz at the potential of the [Fe(CN)_6_]^3−/4−^ redox probe. The amplitude of the alternating voltage was 5 mV.

We modified the surfaces of gold electrodes with d-mannose or mucin, which mimic the outer layer of epithelial cells and the surrounding mucus layer, respectively. Figure 1 presents a schematic of the electrode functionalization with functional groups. A gold electrode on a glass substrate was modified with a monolayer of mercaptoundecanol by self-assembly [26], and this layer was derivatized using epichlorohydrin under basic conditions and subsequent mucin or d-mannose coupling achieved by epoxide ring opening reaction [27]. The surface-functionalized electrode was fixed in microfluidic jig (Figure 2). In this study, we used two-electrode system to simplify the cell setup. The results were not much different from those obtained from three-electrode system. Surface properties of gold electrode at each step were monitored by taking impedimetric measurements in the presence of a [Fe(CN)_6_]^3−/4−^ redox probe. The changes in the electrochemical properties of the redox probe are detailed in the Randle equivalent circuit shown in Figure 2. This equivalent circuit consists of the ohmic resistance of the electrolyte, *R_s_*, the Warburg impedance *Z_w_*, resulting from the diffusion of ions from bulk electrolyte to the electrode, the double layer capacitance, *C*_dl_, and the interfacial electron transfer resistance, *R_et_*. *R_s_* and *Z_w_* are not affected by physicochemical transformations at the electrode surface because they represent bulk properties of the electrolyte solution and diffusion features of the redox probe in solution. On the other hand, *R_et_* and C*_dl_* are determined by dielectric and insulating properties at the electrode/solution interface [28]. The number of bacteria attached to the surface of electrode has been reported to be related with double-layer capacitance [29]. In other words, attached bacteria or any type of attached mass to an electrode would cause a decrease in *C*_dl_ by blocking the electrode surface. Figure 3 shows the Faradaic impedance spectra, presented as Nyquist plots of the gold electrode, over the course of surface modification. Impedance was measured at frequencies ranging from 0.1 to 1 kHz at the formal potential of the [Fe(CN)_6_]^3−/4−^ redox probe. The electron transfer resistance of the gold electrode increased substantially after modifying the surface with a monolayer of mercapto undecanol, which was presumed to be due to the formation of a partitioning or insulating layer of mercapto undecanol on the electrode. The electron transfer resistance of the redox probe further increased after subsequent treatments with epichlorohydrin and mucin. Figure 4 shows a Bode plot and a Nyquist diagram for the Faradaic impedance measurement of a mannose-coated gold electrode in the presence of *K. pneumoniae* 2242 concentrations ranging from 10^5^ to 10^8^ CFU/mL. Impedance changes caused by bacterial attachment to an electrode are typically analyzed using Bode plots [30]. Greatest impedance differences were observed at 0.1 Hz with respect to the control. The number of bacteria attached to the mannose-coated electrode is correlated with double-layer capacitance, which dominates impedance at low frequency, whereas the dielectric capacitance dominates impedance at high frequency.

### 3.2. Adhesion Properties of K. pneumoniae on the Mannose-Modified Surface

**Figure 5:**
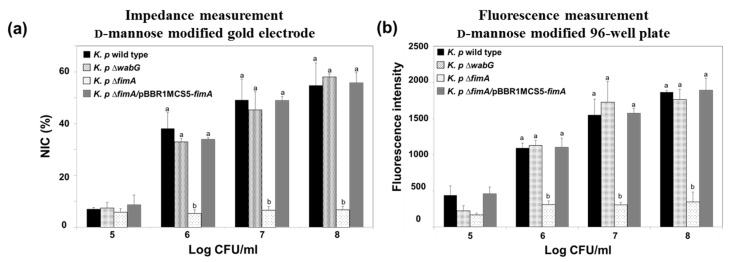
Impedance measurements (**a**) and conventional fluorescence measurements obtained using the 96-well plate method (**b**) for monitoring the adhesion of *K. pneumoniae* KCTC 2242 and its isogenic mutants to the mannose modified electrode. Mean values with different superscripts (a,b) at each bacterial concentration indicate significant differences between the wild type and its isogenic mutant strains (*p* < 0.05).

The fimbriae of *K. pneumoniae* have been reported to be an important adhesion factor as they attach to a specific carbohydrate moiety on the host cell’s surface [17,31]. The fimbriae form a specific interaction with mannosyl residues of N-linked glycoprotein on the outer layer of target cells, which suggests that the fimbriae of *K. pneumoniae* play a critical role in early stage of infection [32]. In this study, we designed a model surface functionalized with mannose on a gold electrode to monitor the presence of *K. pneumoniae* fimbriae using electrochemical impedimetric spectroscopy. The binding ability of *K. pneumoniae* 2242 and of its isogenic mutant, *K. pneumoniae* 2242 ∆*fimA,* which is devoid of fimbriae were also examined using the electrode setup. We also tested the binding characteristics of outer core LPS-truncated mutant strain, *K. pneumoniae* 2242 ∆*wabG*. The *wabG* gene encodes for glucosyltransferase, which is responsible for the attachment of α-l-glycero-d-manno-heptopyranose II to the О-3 position of α-d-galactopyranosyluronic acid. The outer core LPS of *K. pneumoniae* is involved in the formation of a capsule layer on the surface of the bacterium, and thus, the ∆*wabG* mutant lost the ability to form a capsule layer on the surface [21]. The absence of capsule formation by *K. pneumoniae* ∆*wabG* mutant strain was confirmed by microscopic analysis after staining cells with crystal violet and India ink (Figure S1). The binding characteristics of *K. pneumoniae* 2242 and of its isogenic mutants to the mannose-coated electrode were analyzed based on NIC value at 0.1 Hz, which reflects the number of bacteria bound to the surface of the functionalized electrode (Figure 5a). The NIC value was found to increase in proportion to the concentration of test bacteria. The binding pattern of *K. pneumoniae* wild type to the mannose-coated surface was not very different from that of *K. pneumoniae* ∆*wabG*, but the binding ability of *K. pneumoniae* 2242 ∆*fimA* to the mannose surface was significantly reduced at all bacterial concentrations tested, indicating that fimbriae were critically responsible for the attachment of *K. pneumoniae* to mannose surface. To confirm that the loss of the ability of bacteria to bind to the mannose surface was due to the absence of fimbriae, we examined the binding property of the complementary strain, *K. pneumoniae* ∆*fimA*/pBBR1MCS5-*fimA*, to the mannose surface. The binding ability of *K. pneumoniae* ∆*fimA* was fully restored to the level of the wild type by introducing the *fimA* gene to the mutant strain. This results shows that fimbriae were responsible for the attachment of bacteria to the mannose surface. In other words, the binding of *K. pneumoniae* to human epithelial cells is at least in part derived from an interaction between bacterial fimbriae and the mannose moiety in the outer surfaces of epithelial cell.

Impedimetric results were compared with those obtained using a conventional assay conducted using a 96-well plate coated with α-d-mannose. CFDA-labeled *K. pneumoniae* wild type and its isogenic mutants were allowed to bind to the surface of mannose-coated 96-well plates for 1 h. After washing out unbound bacteria from plates with PBS, amounts of bacteria bound to mannose surfaces were assessed by measuring the fluorescence emitted from CFDA-labeled bacteria using a fluorescence reader. The binding patterns of the wild type and its isogenic mutants were almost identical to those obtained from impedimetric measurements (Figure 5b).

**Figure 6:**
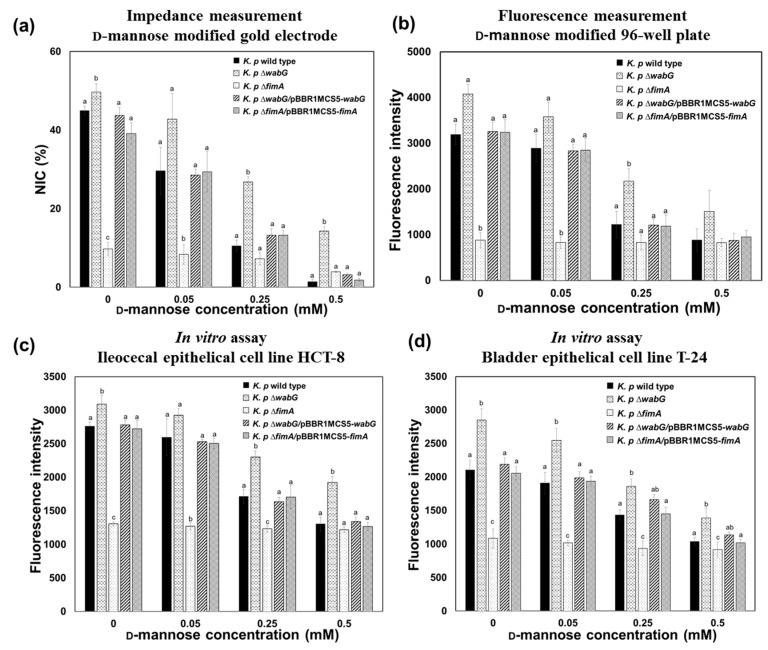
Competition assay results showing the role of mannose on fimbriae-mediated adhesion of *K. pneumoniae* 2242 wild type and its isogenic mutants to the epithelial cells. The results obtained using the impedance sensor (**a**) were compatible with those obtained from the conventional 96-well plate/fluorescence method (**b**) and with in vitro assay results obtained using HCT-8 human ileocecal epithelial cells (**c**) and T-24 bladder epithelial cells (**d**). The ∆*fimA* mutant showed lowest binding to the mannose surface and its binding was unaffected by the presence of free mannose. The concentration of bacteria used for all the test was 10^7^ CFU/mL. Mean values with different superscripts (a,b,c) at each mannose concentration indicate significant differences between the wild type and its isogenic mutant strains (*p* < 0.05).

Assuming that the binding of *K. pneumoniae* to the surface of epithelial cell is due to a specific interaction between fimbriae and mannose moiety of the cell surface, the binding ability of the bacterium should be affected by the presence of free mannose molecules because the free mannose would compete with immobilized mannose on the surface for binding sites on fimbriae. Thus, we carried out a competition assay to confirm this hypothesis and the accuracy of developed impedance sensor. As was expected, binding of *K. pneumoniae* to mannose surfaces was significantly and concentration-dependently inhibited by the presence of free mannose. The binding patterns of *K. pneumoniae* 2242 wild type and its isogenic mutants to the model surface in the absence and presence of varying concentration of free mannose were measured using the impedance sensor, and the results obtained were compared with those obtained using the conventional 96-well plate method (Figure 6a,b). The results obtained using the two methods demonstrated almost the same patterns indicating that the impedance sensor provides accurate and reliable measurements of bacterial interactions with specific surfaces.

The binding properties of *K. pneumoniae* and its inhibition by free mannose were further validated by an in vitro assay using human ileocecal epithelial cells (HCT-8 cells) and bladder epithelial cells (T-24 cells). Binding patterns and degrees of inhibition in the presence of free mannose were consistent with those obtained using the impedance sensor (Figure 6c,d). The binding of *K. pneumoniae* to the mannose-coated surface or to epithelial cells was only affected by the absence of fimbriae in ∆*fimA* mutant strain. Furthermore, the binding abilities of *K. pneumoniae* ∆*fimA* to the mannose surface and to epithelial cells were fully restored to the level of wild type when the *fimA* gene was complemented by introducing pBBR1MCS5-*fimA*. The presence or absence of outer core LPS and capsular polysaccharide did not affect bacterial binding to the mannose surface or to epithelial cells. Interestingly, binding of *K. pneumoniae* 2242 ∆*wabG* mutant to mannose surface and to epithelial cells was even higher than that of its parental strain. This implies that the mutant strain, which lacked both outer core LPS and capsular polysaccharide, is able to associate with epithelial cells more than its parental strain, which is consistent with a previous report that capsular polysaccharide reduces bacterial adherence to epithelial cells in vitro [8,18,33]. In ∆*wabG* mutant, two restraints limit adhesion to epithelial cells. The first is that the thick layer of capsular polysaccharide around bacteria partially masks the adhesion component in fimbriae that otherwise interacts with the mannose moiety of epithelial cells. The second is that capsular polysaccharides interfere with the biosynthesis of fimbrial subunits into mature fimbriae on bacterial surfaces [34]. It is noteworthy that the enhanced binding ability of *K. pneumoniae* 2242 ∆*wabG* to epithelial cells was also clearly monitored by impedimetric measurements of the mannose-coated model surface (Figure 6).

### 3.3. Adhesion Properties of K. pneumoniae on Mucin Modified Surfaces

**Figure 7:**
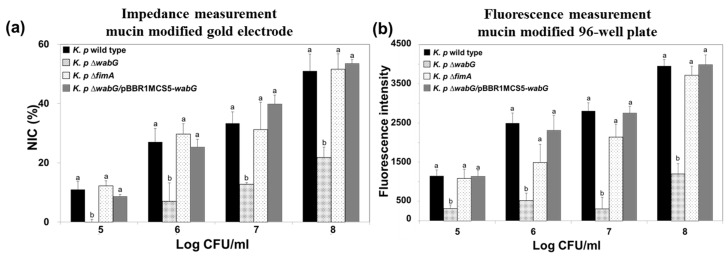
Impedance measurements (**a**) and conventional measurements obtained using the 96-well plate/fluorescence method (**b**) for the adhesion of *K. pneumoniae* KCTC 2242 and of its isogenic mutants to mucin modified surfaces. For impedance measurements, normalized impedance change (NIC) at 0.1 Hz was used to measure the adhesion of bacteria to mucin surfaces. Increases in impedance signals were proportional to number of bacteria in the range from 10^5^ to 10^8^ CFU/mL *K. pneumoniae* KCTC 2242 ∆*wabG* showed the lowest level of interaction with mucin surfaces. Mean values with different superscripts (a,b) at each bacterial concentration indicate significant differences between the wild type and its isogenic mutant strains (*p* < 0.05).

The ability of bacteria to adhere to specific epithelial cells is generally regarded as a potential pathogenic factor during the initial stage of infection. However, it has been reported that the ability of *K. pneumoniae* to adhere to cultured epithelial cells is not necessarily associated with bacterial infectivity, because the results obtained in an in vivo study using mouse model showed no correlation with those of an in vitro study [35]. We suggest that the reason for this results was that most epithelial cells in intestine, lung, and bladder are not directly exposed to lumen but typically are covered with a thick layer of mucous. The mucins are a family of glycoprotein and a major component of the mucous that cover the luminal surfaces of epithelial organs and serves as physical barrier between the extracellular milieu and plasma membrane. Thus, we also functionalized the surface of the gold electrode with mucin to mimic actual luminal surfaces of epithelial organs and to investigate the adhesion properties of *K. pneumoniae* wild type and its isogenic mutants lacking outer core LPS (∆*wabG*) or fimbrial protein (∆*fimA*). The surface of gold electrode was functionalized with porcine gastric mucin which is similar to human colonic mucin. *K. pneumoniae* 2242 wild type or its isogenic mutant strains were introduced to the chamber equipped with a mucin-coated electrode and allowed to adhere to its surface. After washing the surface with PBS, amounts of bound bacteria on mucin surfaces were measured using the impedance sensor. Increases in NIC values indicated an association between bacteria and the artificial mucous layer on the gold electrode, and are proportional to the number of bound bacteria (Figure 7). It is interesting to note that adhesion of bacteria to the artificial mucous layer was unaffected by the absence or presence of fimbriae but was affected by outer core LPS and capsular polysaccharide. Adhesion level to the artificial mucous layer was significantly lower for *K. pneumoniae* ∆*wabG* mutant than for the wild type. In addition, the binding ability of *K. pneumoniae* ∆*wabG* mutant to the mucous layer was fully restored by complementing ∆*wabG* mutant strain with pBBR1MCS5-*wabG* plasmid, which implied that the association between bacteria and the mucous layer was mediated through outer-core LPS or capsular polysaccharide on the outer surface of the bacterium.

The association between *K. pneumoniae* and the mucous layer was also investigated using a conventional 96-well plate coated with mucin, and the results obtained were compared with those obtained using the impedance sensor. We found the results were almost identical, which indicated the impedance measurements offer a reliable alternative to the conventional staining method.

## 4. Conclusions

A simple and sensitive impedance sensor was developed to monitor the major pathogenic factors of *K. pneumoniae*. The ability of *K. pneumoniae* to adhere to host cells is governed by fimbriae, outer core LPS, and capsular polysaccharide, which have specific affinities for the d-mannose-containing trisaccharides of the host glycoprotein and the mucous layer that covers epithelial organs. We designed an electrode functionalized with mannose or mucin to mimic epithelial cell surfaces and the luminal surfaces of epithelial organs. The binding characteristics of *K. pneumoniae* 2242 and its isogenic mutants, ∆*fimA* and ∆*wabG*, which lacked fimbriae and outer-core LPS, respectively were examined using an impedance sensor equipped with a functionalized electrode. Based on the results obtained, we conclude that type 1 fimbriae are responsible for the recognition of and binding to mannose containing trisaccharides of host glycoprotein, whereas outer-core LPS and capsular polysaccharide of bacteria are associated with bacterial interactions with mucins. Wild type *K. pneumoniae* 2242 demonstrated strong interactions with mannose and mucin-coated surfaces based on impedance measurements. However, the binding ability of the bacterium to mannose surfaces was significantly diminished when the *fimA* gene was knocked out. Binding of the bacterium to mucin surfaces was also significantly hampered by removing the ∆*wabG* gene, which is responsible for the synthesis of outer-core LPS. Furthermore, based on impedance measurements, the binding abilities of these knockout variants were fully restored by complementing the mutant strains with the *fimA* or *wabG* gene, respectively, which suggested these genes are responsible for specific interactions. In addition, impedance results were compared with those obtained using the conventional 96-well plate method and in vitro assays using HCT-8 human ileocecal epithelial cells or T-24 human bladder epithelial cells. We found all impedance measurements were consistent with conventional and in vitro assay results, which indicates that the devised impedance sensor implemented using a functionalized model surface could replace conventional methods. We suggest this impedance sensing system offers a promising alternative means of monitoring or investigating the virulence factors of pathogenic bacteria associated with detrimental interactions with specific tissue or organs at early stages of infections.

## Supplementary Materials

The following are available online at http://www.mdpi.com/1424-8220/17/6/1406/s1, Figure S1: Visualization of capsule in *K. pneumoniae* KCTC 2242 and its isogenic mutants.
