# Supplementary material for: Identification of Pathogenic Factors in Klebsiella pneumoniae Using Impedimetric Sensor Equipped with Biomimetic Surfaces
Source: Sensors (Basel). 2017 Jun 15;17(6):1406. doi: 10.3390/s17061406 (PMC5492845; doi:10.3390/s17061406)
Supplement: Supplementary file 1 [file sensors-17-01406-s001.pdf]

**Supplementary Material**

# Identification of Pathogenic Factors in *Klebsiella pneumoniae* Using Impedimetric Sensor Equipped with Biomimetic Surfaces

Duyen Thi Ngoc Huynh, Ah-Young Kim, Young-Rok Kim*

Department of Food Science and Biotechnology & Graduate School of Biotechnology,

College of Life Sciences, Kyung Hee University, Yongin, 17104, Korea.

* Corresponding Authors: youngkim@khu.ac.kr

Tel: +82-31-201-3830; Fax: +82-31-204-8116

**Capsule staining**

*K. pneumoniae* and its isogenic mutants were stained with crystal violet and India ink to visualize the presence of capsule. Overnight cultures of *K. pneumoniae* cells were mixed with 4 µl india ink and spread on slide glass to form a thin film. To prevent destruction of the bacterial capsules from heat, the samples were air-dried at room temperature for 15 min. The samples were then stained with 0.1 % crystal violet for 1 min and washed with distilled water to remove unbound crystal violet. After drying at room temperature the slide glasses were examined under inverted bright field microscope (Nicon TE200, Tokyo, Japan).

The absence of capsule in *K. pneumoniae* 2242*ΔwabG* mutant was confirmed by the light microscope after staining the bacteria with crystal violet. The bacteria that do not produce capsule is stained by crystal violet and seen as pink, whereas the one with capsule is shown as white layer around the cell because the capsule surrounding the bacteria prevents the free diffusion of dye molecules to the cell membrane [1, 2]. The parental strain, *K. pneumoniae*

2242, *K. pneumoniae* 2242*ΔfimA* mutant strain, and *wabG*-complemented strain *K. pneumoniae* 2242*ΔwabG*/pBBR1MCS5-*wabG* were found to have a capsule surrounding the bacteria (Fig S1-a, c, d). On the other hand, the capsule is absent in *K. pneumoniae* 2242*ΔwabG* mutant of which the gene encoding outer core LPS is knocked out (Fig S1-b).

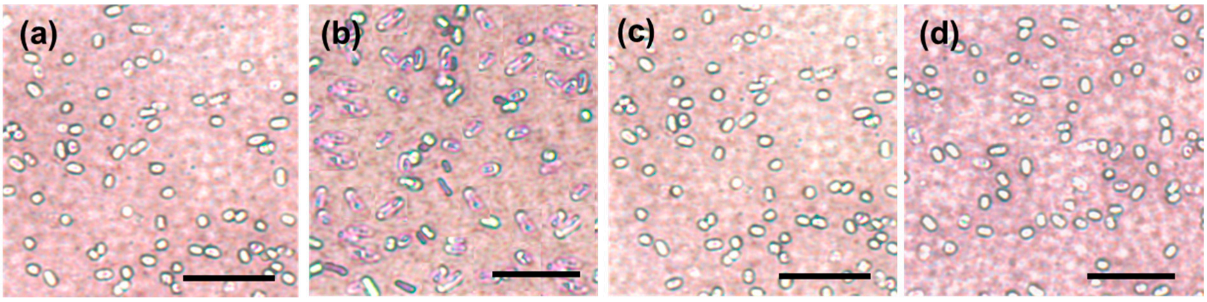


**Fig. S1**. Visualization of capsule in *K. pneumoniae* KCTC 2242 (a), *K. pneumoniae* 2242 *ΔwabG* mutant (b), *K. pneumoniae* 2242 *ΔfimA* mutant (c), and *K. pneumoniae* 2242 *ΔwabG*/pBBR1MCS5-*wabG* (d) after negative capsule staining with crystal violet and India ink. The capsules are shown in white layer around the bacterial surface in (a), (c) and (d). *K. pneumoniae* 2242 *ΔwabG* mutant is shown in pink, suggesting the absence of capsule (b). *Scale bar* is 5 μm.

**Reference**


1. Izquierdo, L.; Coderch, N.; Piqué, N.; Bedini, E.; Corsaro, M. M.; Merino, S.; Fresno, S.; Tomás, J. M.; Regué, M., The *Klebsiella pneumoniae wabG* gene: role in biosynthesis of the core lipopolysaccharide and virulence. *J. Bacteriol* **2003,** 185, (24), 7213-7221.
2. Jung, S.-G.; Jang, J.-H.; Kim, A.-Y.; Lim, M.-C.; Kim, B.; Lee, J.; Kim, Y.-R., Removal of pathogenic factors from 2, 3-butanediol-producing *Klebsiella* species by inactivating virulence-related wabG gene. *Appl. Microbio. Biotechnol* **2013,** 97, (5), 1997-2007.
